# Radiopaque Chitosan Ducts Fabricated by Extrusion-Based 3D Printing to Promote Healing After Pancreaticoenterostomy

**DOI:** 10.3389/fbioe.2021.686207

**Published:** 2021-06-04

**Authors:** Maoen Pan, Chaoqian Zhao, Zeya Xu, Yuanyuan Yang, Tianhong Teng, Jinxin Lin, Heguang Huang

**Affiliations:** ^1^Department of General Surgery, Fujian Medical University Union Hospital, Fuzhou, China; ^2^Key Laboratory of Optoelectronic Materials Chemical and Physics, Fujian Institute of Research on the Structure of Matter, Chinese Academy of Sciences, Fuzhou, China

**Keywords:** 3D printing, chitosan, pancreatic duct stent, biocompatible, degradation

## Abstract

Long-term placement of non-degradable silicone rubber pancreatic duct stents in the body is likely to cause inflammation and injury. Therefore, it is necessary to develop degradable and biocompatible stents to replace silicone rubber tubes as pancreatic duct stents. The purpose of our research was to verify the feasibility and biological safety of extrusion-based 3D printed radiopaque chitosan (CS) ducts for pancreaticojejunostomy. Chitosan-barium sulfate (CS-Ba) ducts with different molecular weights (low-, medium-, and high-molecular weight CS-Ba: LCS-Ba, MCS-Ba, and HCS-Ba, respectively) were soaked *in vitro* in simulated pancreatic juice (SPJ) (pH 8.0) with or without pancreatin for 16 weeks. Changes in their weight, water absorption rate and mechanical properties were tested regularly. The biocompatibility, degradation and radiopaque performance were verified by *in vivo* and *in vitro* experiments. The results showed that CS-Ba ducts prepared by this method had regular compact structures and good molding effects. In addition, the lower the molecular weight of the CS-Ba ducts was, the faster the degradation rate was. Extrusion-based 3D-printed CS-Ba ducts have mechanical properties that match those of soft tissue, good biocompatibility and radioopacity. *In vitro* studies have also shown that CS-Ba ducts can promote the growth of fibroblasts. These stents have great potential for use in pancreatic duct stent applications in the future.

## Introduction

Pancreaticoduodenectomy (PD) is still the classic method of treatment of benign and malignant tumors of the pancreas, ampullary and duodenum. However, the incidence of postoperative complications is still higher than 30–40% due to complicated procedures (Gouma et al., 2000; Bassi et al., 2001). Pancreaticojejunostomy is the most important and complicated procedure in PD surgery. Pancreatic fistula is the most serious complication after pancreaticojejunostomy (Karim et al., 2018). Severe pancreatic fistula can cause abdominal infection and hemorrhage, and even progress to severe sepsis. Therefore, it is recommended to use pancreatic duct stents to reduce complications and mortality during PD and middle segment pancreatectomy. To drain pancreatic fluid, placement of a silicone rubber stent into the main pancreatic duct has been proven to be an ideal method to promote anastomotic healing by preventing trypsin from corroding the anastomosis in the early postoperative stage and by reducing the incidence of postoperative pancreatic fistula (Isayama et al., 2016). However, as these materials are non-degradable and easily cause inflammation after long-term use, their applications are limited. Some studies have used polylactic acid (PLA) to prepare pancreatic duct stents (Yang et al., 2019). However, the high modulus of this material, which is not consistent with that of soft tissue, is prone to induce damage and inflammation during its use, and it takes a long time to degrade. Therefore, it is necessary to develop a material with a suitable degradation time and Young’s modulus for the preparation of pancreatic duct stents.

Various methods have been used to prevent anastomotic fistula, but the study results show that they produced little difference in the rate of pancreatic fistula, and the incidence of B-grade and C-grade pancreatic fistula is still as high as 19.8% (Peng et al., 2002; Bassi et al., 2017). Therefore, it is of great significance to reduce the incidence of early pancreatic fistula by developing a new pancreatic duct stent material. Traditional silicone rubber tubes are inert materials and have no biological activity. However, with the increasing application of natural biodegradable materials in clinical practice, this shortcoming can be effectively solved.

Chitosan (CS), which is the deacetylated form of chitin (degree of deacetylation >50%), is a naturally occurring polymer and the second most abundant natural biopolymer after cellulose (Ahmed and Ikram, 2016). CS is an excellent biocompatible polymer with a range of properties, such as non-toxicity, biodegradability, antimicrobial and immune-modulatory activities (Anitha et al., 2014). Most importantly, CS has the potential to promote soft tissue healing (Ahmed and Ikram, 2016). Our previous research (Zhao et al., 2020) studied the preparation of extrusion-based 3D printing of CS ducts for soft tissue engineering. It was found that the mechanical properties of these CS ducts matched well with those of soft tissue and had great potential in soft tissue engineering.

When a biodegradable stent is developed, it is essential to know how long it will take to degrade. This means that we must be able to track its degradation *in vivo*. In future clinical applications, postoperative follow-up observation of patients is very important. X-ray imaging technology is an effective and convenient method for detecting and evaluating the position and shape of implant materials (Sang et al., 2014; Houston et al., 2017). Traditional polymer molecules should be endowed with radiopaque properties to meet medical requirements. Barium sulfate is the common imaging agent used to enhance the imaging effects of polymers in the body and facilitate the dynamic observation of implant material changes after surgery.

It is very difficult to prepare the complicated structures of ducts by traditional manufacturing technologies, whereas the emergence of 3D printing provides vast opportunities for personalized medicine (Shi and Huang, 2020). In this study, based on clinical requirements, radiopaque pancreatic duct stents using degradable CS and barium sulfate were prepared by extrusion-based 3D printing technology for the first time. To evaluate the mechanical matching of CS-BaSO_4_ (CS-Ba) to pancreatic tissue, the mechanical properties of CS-Ba were tested during its degradation in simulated pancreatic juice (SPJ). The biosafety and applicability of the CS-Ba composites were evaluated by biocompatibility testing *in vitro* and *in vivo*. In addition, the radiopaque effects of CS-Ba ducts were studied *in vivo*. Overall, this study will verify the advantages of extrusion-based 3D-printed CS-Ba ducts as pancreatic duct stents in many aspects.

## Experimental

### Materials

Three CS samples (deacetylation: 95%) with different molecular weights, including low molecular weight (50,000 g/mol, LCS), medium molecular weight (200,000 g/mol, MCS), and high molecular weight (500,000 g/mol, HCS) CS, were purchased from Cool Chemical Science and Technology Co., Ltd., China. Barium sulfate (BaSO_4_, 99%), glycolic acid (GA) (C_2_H_4_O_3_, 98%) (GA), potassium hydroxide (KOH, 85%), sodium chloride (NaCl, 99.5%), and the analytical reagent ethanol (C_2_H_6_O, 99.7%) were purchased from Sinopharm Chemical Reagents Co., Ltd., China. Additionally, pancreatin from porcine pancreas was purchased from Sigma-Aldrich, St. Louis, MO, United States; the Live-Dead Cell Staining Kit was from Dalian Meilun Biotechnology Co., Ltd., China; the mouse TNF-α/IL-10 Quantikine ELISA Kit was purchased from R&D Systems, United States; and the DNA Content Quantitation Assay Kit (Cell Cycle) and lipopolysaccharide (LPS) were purchased from Beijing Solarbio Science & Technology Co., Ltd., China. Mouse fibroblasts (L929 cells) and mouse RAW264.7 macrophages were purchased from Shanghai Cell Bank, Chinese Academy of Sciences, and Sprague-Dawley (SD) rats were purchased from Experimental Animal Center, Fujian Medical University.

Preparation of the solution of artificial SPJ was performed according to Charteris et al. (1998) and Del Piano et al. (2008). A solution containing 0.1% pancreatin and 0.5% sodium chloride was prepared using deionized water, and the pH was adjusted to 8.0 by the addition of NaOH.

### Duct Preparation

**FIGURE 1:**
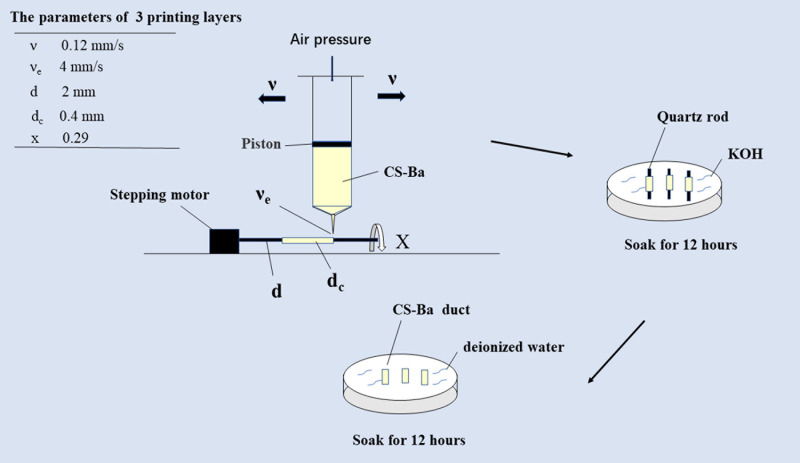
Schematic diagram of extrusion-based 3D-printed CS-Ba ducts combined with the rotary axis.

CS-Ba ducts were prepared with a weight mixing ratio of 23% barium sulfate to CS with different molecular weights. GA was added for dissolution at a ratio of solute to solvent of 1:4, and the mixture was stirred evenly. Combination with rotation-axis and slurry-extrusion 3D printing (cRS3DP) was used to fabricate CS-Ba ducts. A stepping motor was used to drive the quartz rod to match the speed. In this study, the 3D printing parameters were as follows: the printing layer (*d*_*c*_) was 0.4 mm in each layer, the printing speed (υ_*e*_) was 4 mm/s, and the air pressure was 0.8 MPa, the diameter of the rotary axis (*d*) was 2 mm, the moving speed of the syringe needle (υ) was 0.12 mm/s and the rotary speed of the rotary axis (*x*) was 0.29 r/s (Zhao et al., 2020). The number of printing layers was adjusted according to the required diameter of the ducts. The duct cavity diameter was determined by the diameter of the quartz rod. The function of the quartz rod was to act as a rotary axis driven by a stepper motor for slurry deposition. The printed quartz rod and CS-Ba ducts were soaked in KOH for 12 h and then transferred to deionized water for 12 h. Finally, the quartz rod in the middle of the ducts were removed, and the shaped ducts were stored in deionized water for later use (diagram shown in Figure 1). Because the quartz rod is smooth, it is easy to remove from the ducts.

### X-ray Diffraction (XRD) Patterns and Fourier Transform Infrared Spectroscopy of CS-Ba Ducts

The sample was placed on a sample stage for analysis. A Cu target, a tube voltage of 40 kV, a tube current of 5 mA, continuous scanning at 2θ = 5–85°, a scanning speed of 5°/min, and a step size of 0.02° were used. Fourier transform infrared (FTIR) was used to characterize functional groups with the following parameters: infrared scanning range, wavelength range of 4,000–650 cm^–1^, resolution of 4 cm^–1^, and 16 scans.

### *In vitro* Swelling and Degradation Tests of the CS-Ba Ducts

Different molecular weights of CS mixtures (LCS-Ba, MCS-Ba, and HCS-Ba) (*n* ≥ 15 per group) were accurately weighed after drying at 65°C for 24 h and soaked in SPJ solution. The ratio of mass to solvent was 1:30 g/mL, and the solution was changed every other day to maintain the activity of pancreatin. At 2, 4, 8, 12, and 16 weeks, three samples from each group were removed and weighed after washing and drying at 65°C for 24 h. The degradation rate was calculated as the degradation rate = [(*W*_0_−*W*′)/*W*_0_]×100% (where *W*_0_ is the original weight and *W’* is the dried weight). To observe the degradation of CS-Ba by pancreatin in this study, an SPJ solution without pancreatin was created (with the other conditions remaining the same), and the degradation rates of the samples in the SPJ solutions were compared.

To evaluate the changes in the water swelling rates of the samples during the degradation process, samples from different groups were removed at 4, 8, and 16 weeks, and after the water on the surface of the samples was wiped dry with filter paper, *W*_0_ was recorded. The samples were placed in an oven and weighed upon reaching a constant weight to determine *W*_d_. The swelling percentage was calculated as follows: S% = [(*W*_0_−*W*_d_)/*W*_0_]×100%.

### Mechanical Strength Measurements of the CS-Ba Rods

The samples were made into cylindrical tensile samples with cross sections approximately 1.5 mm in diameter. A universal testing machine (CMT4304 and SANS) with a tensile speed of 10 mm/s was used to test each sample’s original tensile strength, Young’s modulus and fracture stress according to ISO 527-1 2012. Samples were soaked in the alkaline SPJ solution. The mechanical properties of the samples were tested at 4, 8, and 16 weeks and compared with the original mechanical properties to observe the mechanical strength changes during the degradation process.

### CS-Ba Duct Cell Viability Assays

The MTT method was used to test the *in vitro* cytotoxicity of different molecular weight of samples. Extraction of the sample: According to ISO 10993-12:2012 (E), the extraction ratio was 0.2 g/mL, the extraction medium was modified Eagle medium (MEM) medium containing 10% fetal bovine serum (FBS), the extraction temperature was (37 ± 1)°C, and the extraction time was (24 ± 2) h. The blank control group used MEM containing 10% FBS, the negative control group was a high-density polyethylene (HDPE) extract group, and the positive control was a complete medium containing 0.64% phenol. The absorbance was measured at 570 nm with a microplate reader, and the survival rate (%) was calculated according to the following formula:

$$\mathrm{Cell}\mathrm{viability}\left(\%\right)=\left({\text{OD}}_{570\,\text{e}}/{\text{OD}}_{570\,\text{b}}\right)\times 100\%,$$

where OD_570__*e*_ is the absorbance of the 100% extract and OD_570__*b*_ is the absorbance of the blank group. If the cell survival rate is less than 70% that of the blank cells, the compound is potentially cytotoxic.

### Live/Dead Fluorescence Staining

One hundred microliters of L929 cells were inoculated into a 96-well plate at a density of 1 × 105 cells/mL per well. The cells were incubated with complete medium for 24 h, and then the complete medium was replaced with the corresponding sample extract. After incubation for 24 or 48 h, the extract was discarded and the cells were washed with PBS twice. The dyes calcein-AM and propidium iodide (PI) were added followed by incubation at 37°C in the dark for 30 min. The staining solution was discarded, and 100 μL of PBS was added. The morphology and viability of the cells were observed under an inverted fluorescence microscope. Green fluorescence indicated living cells, and red fluorescence indicated dead cells.

### Cell Cycle Detection

L929 cells were cultured with extracts of the three kinds of ducts (The extraction method of sample extract was shown in section “CS-Ba Duct Cell Viability Assays”), and the control group was cultured with complete medium. After 24 h, the cells were digested with 0.25% trypsin to prepare a single cell suspension, which was fixed with 70% ice ethanol for 24 h. After centrifugation at 1000 rpm, the cells were washed with PBS twice and incubated at 37°C for 30 min after the addition of 0.25 mg/mL RNase. Next, PI solution was added followed by incubation at 4°C for 30 min. The supernatant was discarded after centrifugation, and 500 μL of PBS was added. Cell cycle changes were measured with a BD Accuri C6 Plus cytometer (United States).

### Hemolysis Assay

Rat blood (1 mL) was centrifuged for 10 min at 3000 rpm, the upper serum was discarded, and the samples were washed three times with PBS. Then, 20 μL of red blood cells were treated by the addition of 500 μL of PBS extract with different molecular weights of CS-Ba samples, which were exposed to ionizing water and PBS as positive and negative controls, respectively, for 4 h at 37°C. The absorption sensitivity of the hemoglobin in the liquid was monitored with an ultraviolet spectrometer after centrifugation for 10 min at a speed of 3000 rpm, and the hemolysis rate (HR) of the solution was calculated using the following formula. In the formula, *A*_*t*_, *A*_*pc*_, and *A*_*nc*_ are the absorbance of the supernatant at 540 nm for the test sample and the positive and negative controls, respectively. HR(%) = (*A*_t_−*A*_nc_)/(*A*_pc_−*A*_nc_)×100%.

### Measurement of the Production of Inflammatory Factors *in vitro*

Mouse RAW 264.7 macrophages were cultured in Dulbecco’s modified Eagle medium (DMEM) containing 10% FBS. The concentration of the cells was adjusted to 1 × 10^5^/mL in complete culture medium. The cells were inoculated into 96-well plates at 100 μL per well. After 24 h of culture, the original culture medium in the wells was discarded. The negative control group was supplemented with 100 μL of complete culture medium, and the positive control group was supplemented with complete culture medium containing 1 μg/mL LPS. Then, the LCS-Ba, MCS-Ba, and HCS-Ba samples were added to the extract solution. The supernatant of the cell culture was extracted 24 h later, and the TNF-α and IL-10 contents were determined with ELISA kits.

### Animal Experiment

The animal research protocol was approved by the Experimental Animal Ethical Committee of Fujian Medical University (No: 2020-0022). Thirty-two SD rats weighing 200–250 g were randomly divided into four groups. The control group was implanted with inert silicone rubber tubes, and the other groups were implanted with LCS-Ba, MCS-Ba, and HCS-Ba ducts. Each duct was placed in the abdominal cavity and fixed to the surface of the pancreas with 5–0 non-absorbable sutures (as shown in Figure 4A). Serum amylase was then measured in blood from the tail vein of three rats in each group 1 day before surgery and 1, 3, and 7 days after surgery. Eight and sixteen weeks after the operation, the radiopaque performance and degradation of the CS-Ba ducts in each group of rats were observed by X-ray, and venous blood was taken for the detection of liver and kidney function. Then, four rats from each group were euthanized, and the pancreatic tissues around the ducts were taken as pathological sections to observe the inflammation of the tissues around the ducts. During the 16th week, the hearts, liver, spleen, lungs and kidneys of the rats were taken for hemoxylin-eosin (HE) staining to observe the toxicity of the test materials *in vivo* after long-term implantation. Finally, the removed ducts were cleaned and dried to a constant weight, and the duct degradation rate was determined after comparison with the weight before implantation. The surface morphologies of the materials were observed by scanning electron microscopy (SEM).

**FIGURE 2:**
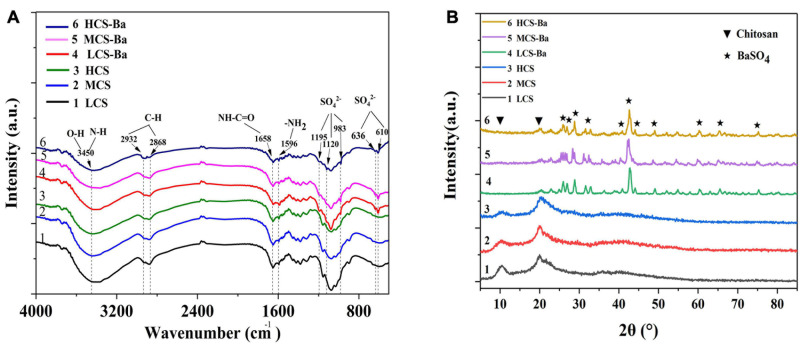
Comparison of the **(A)** FTIR spectra and **(B)** XRD patterns of CS-Ba and CS ducts with different molecular weights.

**FIGURE 3:**
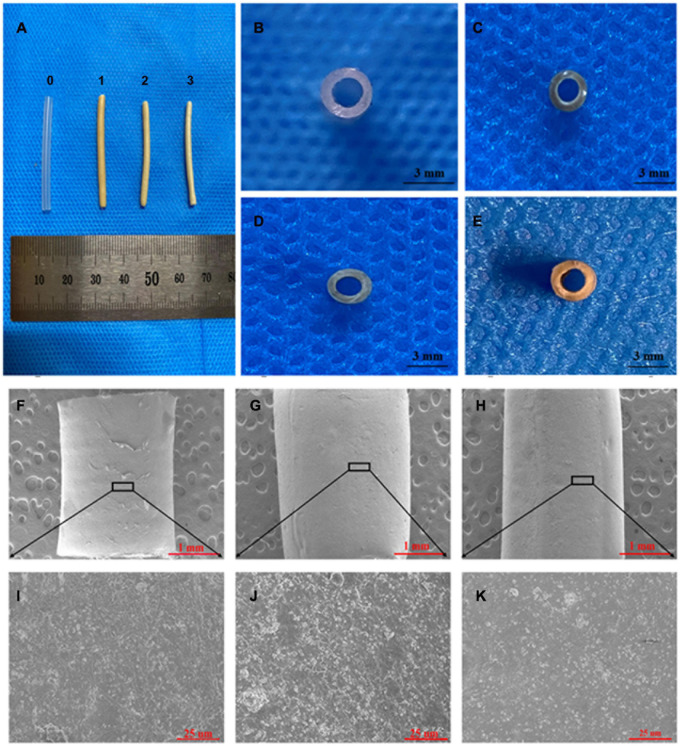
SEM and visual observations of the CS-Ba ducts prepared by extrusion-based 3D printing. **(A, C–E)** Macro morphologies of the LCS-Ba, MCS-Ba, and HCS-Ba samples in different positions (0-silicone rubber, 1-LCS-Ba, 2-MCS-Ba, and 3-HCS-Ba). **(B)** A silicone rubber tube was used as a control. **(F–K)** Radial micromorphology of the LCS-Ba **(F)**, MCS-Ba **(G)**, and HCS-Ba **(H)** ducts.

**FIGURE 4:**
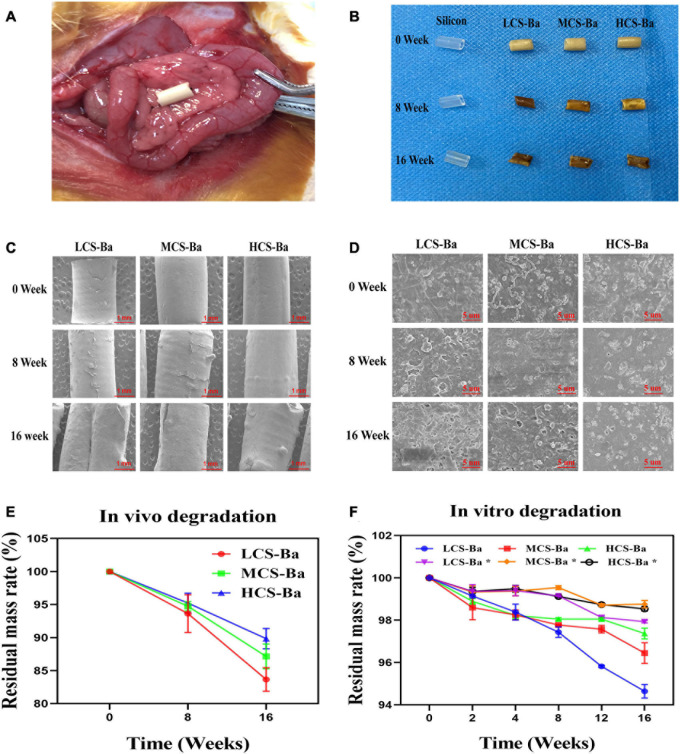
**(A)** Ducts were fixed on the surface of the rat pancreas. **(B)** Physical image of the original stent and the implanted ducts at 8 and 16 weeks. **(C, D)** SEM images of the changes in the microstructures of the original ducts and the implanted ducts at 8 and 16 weeks (scales are 1 mm and 5 μm, respectively). **(E)** *In vivo* degradation rate. **(F)** The degradation rates of different molecular weights of CS-Ba ducts *in vitro* (* SPJ solution without pancreatin).

### Statistical Analysis

All experiments were repeated three or more times (*n* ≥ 3) and the data are presented as mean ± SD. Statistical analysis was performed using one-way ANOVA, and *P* < 0.05 was considered statistically significant.

## Results and Discussion

Due to the limitations of biodegradable materials, many stents that have been researched have a long degradation time and high Young’s modulus, which restricts their scope of clinical use (Parviainen et al., 2000; Laukkarinen et al., 2008; Nordback et al., 2012). Therefore, it is necessary to constantly explore new materials and develop pancreatic duct stents that are more suitable for clinical needs. As a natural biopolymer material, CS has been widely studied and used in various disciplines due to its excellent biological properties (Griffon et al., 2005; Biazar and Heidari Keshel, 2014; Dan et al., 2016). However, there are few studies on its use as a pancreatic duct stent. Based on the above experimental results, this study will verify the feasibility and safety of CS as a pancreatic duct stent from multiple aspects, such as its degradation performance, mechanical properties and biocompatibility.

### Preparation and Identification of CS-Ba Ducts

The preparation of 3D CS ducts is currently difficult. Due to the characteristics of CS, it cannot be 3D printed by thermal processing, and CS ducts prepared by the freeze-drying method are not suitable for pancreatic duct stents due to their irregularities and large porosity (Ao et al., 2006; Nawrotek et al., 2016). Irregular and large pores may cause pancreatic fluid to leak from the lateral wall of the catheter and thus corrode the surrounding anastomotic tissue, causing anastomotic damage. Therefore, CS ducts were prepared by 3D printing by extrusion molding and it was verified that the use of GA to dissolve CS can achieve the best biocompatibility (Zhao et al., 2020). To implant CS in the body and obtain good visualization, we added barium sulfate to the CS to prepare a slurry. The infrared spectra of CS and different molecular weights of CS-Ba samples are shown in Figure 2A. The main functional groups of CS included NH_2_ (1596 cm^–1^), C = O-NHR (1658 cm^–1^), axial C-H (2868, 2922 cm^–1^), and −OH and N-H (3400 cm^–1^) (Barbosa et al., 2019). The characteristic peaks of SO_4_^2–^ were located at 1195, 1120, and 983 cm^–1^, corresponding to symmetrical vibrations, and the out-of-plane vibrations of SO_4_^2–^ at to 610 and 636 cm^–1^ (Sivakumar et al., 2015). The CS-Ba ducts prepared in this study had obvious vibration peaks in both places, indicating that the synthesized product contained barium sulfate particles. In addition, comparison of the spectra of different molecular weights of CS showed that the basic skeleton structure of the CS molecules did not change, indicating that the molecular weight had little effect on the chemical structure. Figure 2B shows the XRD patterns of CS and CS-Ba with different molecular weights. The spectrum of pure CS showed two characteristic diffraction peaks at 11.40 and 20.20°. These are the characteristic peaks of the semicrystalline structure of CS (Samanta and Ray, 2014). The diffraction peaks from CS-Ba at 2θ = 25.8, 26.8, 28.7, 32.8, 41.6, 42.6, 45.9, 48.2, 60.8, 65.8, and 75.8°correspond to the barium sulfate standard diagram (210), (102), (211), (020), (022), (113), (411), (402), (232), (224), and (125) crystal planes (PDF#83-2053). The presence of these peaks indicated an increase in local crystallinity.

As shown in Figure 3, the color of the extrusion-based 3D printed CS-Ba ducts was light yellow, and there was no significant difference in the color between the different molecular weights of CS-Ba ducts. The CS-Ba duct surfaces were relatively smooth, their shapes were regular, and they had good flexibility after the absorption of water and subsequent expansion. In the dry state, the duct walls became hard, and hollow ducts had good patency from the side view (Figures 3A–E). Electron microscopy observations showed that the surfaces of the ducts prepared by the extrusion method were uniform and flat with dense structures and little particle deposition (Figures 3F–K). It was found that the CS-Ba ducts prepared by this method had a dense structure with regular microfibers on the surface and few holes. This dense structure can prevent pancreatic juice from leaking from the wall to the outer side and corroding anastomotic tissue. The microstructure confirmed that the CS-Ba ducts prepared by this method were suitable for use as pancreatic duct stents during pancreaticojejunostomy.

### Degradation of CS-Ba in SPJ and *in vivo*

The ideal biomaterial needs a degradation rate that matches the rate of tissue regeneration to ensure normal healing of the anastomosis after pancreaticojejunostomy while avoiding complications such as anastomotic stenosis caused by rapid degradation of the stents. The most optimal degradation time of the pancreatic duct stent is approximately 3–6 months. Enzymatic hydrolysis is one of the important ways of chitosan degradation *in vivo*. In addition to specific enzymes such as chitinase, chitosanase, and lysozyme, chitosan can also be degraded by non-specific enzymes such as cellulase, lipase, pepsin and papain (Lee et al., 2008; Suwan et al., 2009; Pan et al., 2016). Lysozyme is the main chitosan degrading enzyme in the human body. However, pancreatin is the enzyme that is directly contact with the CS-Ba ducts. In this study, we compared the degradation of CS-Ba ducts in solutions both with and without pancreatin (Figure 4F). The results showed that the degradation of the three types of CS-Ba ducts in the SPJ solution containing pancreatin was significantly faster than that of the ducts in the SPJ solution without pancreatin (*P* < 0.05). In the SPJ solution containing pancreatin, the degradation rate of the LCS-Ba ducts was significantly faster than that of the MCS-Ba and HCS-Ba ducts (*P* < 0.05). The environment of human pancreatic juice is weakly alkaline (pH 7.8–8.4). Under weakly alkaline conditions, the digestive effects of pancreatin are the strongest. In a pH > 6 environment, the dissolution rate of CS is reduced due to the deprotonation of the amino groups present (Matica et al., 2019). Therefore, under the combined effects of many factors, the SPJ solution containing pancreatin can degrade CS-Ba ducts faster than the SPJ solution without pancreatin. In addition, the molecular weight is an important factor that affects the degradation of CS—the lower the molecular weight is, the faster the degradation rate will be (Varoni et al., 2018). Therefore, the molecular weight of CS can be adjusted to meet the degradation time requirement of pancreatic duct stents.

Figure 4E displays the mass losses of the CS-Ba ducts after implantation. Compared with the original masses, the average mass losses of LCS-Ba, MCS-Ba, and HCS-Ba after 8 weeks of implantation were 6.37, 5.25, and 4.77%, respectively, and the average mass losses after 16 weeks were 16.34, 12.85, and 10.16%, respectively. We found that the degradation rate of LCS-Ba was significantly faster than the rates of MCS-Ba and HCS-Ba (*P* < 0.05). Moreover, the degradation rates of the different molecular weights of CS-Ba ducts were found to be significantly faster *in vivo* than *in vitro*, which was related to the wet environment inside the body and the acidic nature of cellular metabolites. The body temperature and lysozymes also promoted the degradation of the materials. As shown in Figure 4B, images of the ducts after degradation showed that the color became black, the texture became brittle, and part of the ducts appeared to be deformed and broken. The ducts at different time points were observed by SEM, and very small scaffold fragments and etched cavities appeared on the surface of each group at 8 weeks. With further degradation at 16 weeks, the surface cracks of the ducts deepened, parts of the ducts collapsed and the etched cavity increased. The changes in the LCS-Ba ducts were the most obvious among all of the ducts (Figures 4C,D). In addition, during degradation in the body, the surface of the material gradually turned from pale yellow to black. This phenomenon is very similar to the darkening of CS after heat treatment, radiation treatment or the Maillard reaction (Yue, 2014; Shamekhi et al., 2017). The essence of the Maillard reaction is the condensation reaction between carbonyl ammonia. Since most of the enzymes in the body are proteins, their carboxyl group and the amino group of chitosan may react under certain conditions to produce the complex black polymer protein melanin, leading to blackening of the surface of the ducts.

### *In vitro* and *in vivo* Biocompatibility of CS-Ba Ducts

**FIGURE 5:**
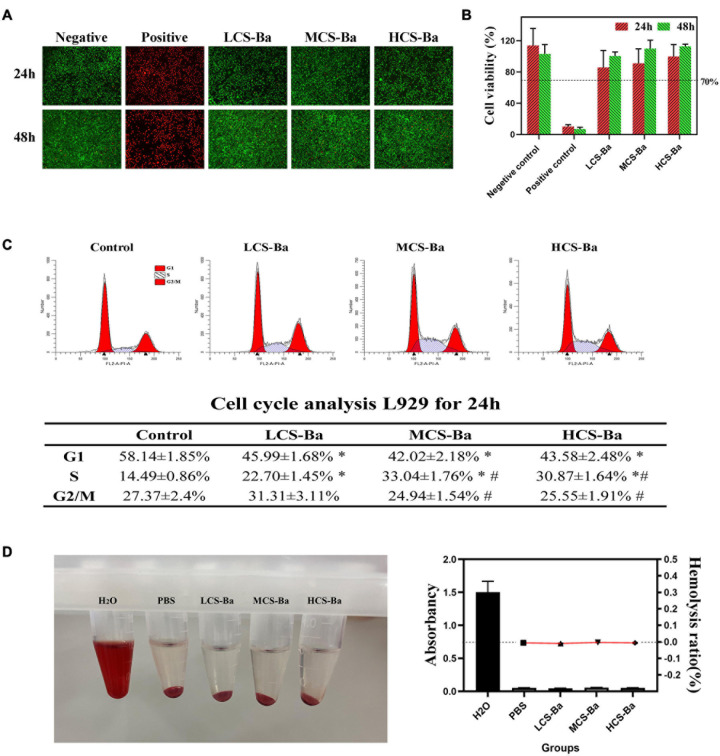
Cytotoxicity and blood compatibility of CS-Ba ducts. **(A)** Live-dead fluorescence staining and **(B)** cell viability of CS-Ba ducts with original extract solutions. **(C)** Effects of CS-Ba duct extraction solution on the L929 cell proliferation cycle. **(D)** The hemolysis rate of CS-Ba ducts (^∗^ represents a significant result compared with the control group, *P* < 0.05; ^#^ represents significant results compared with the LCS-Ba group, *P* < 0.05).

Any medical device applied to the human body must undergo toxicity testing before clinical application. Chitosan has been proven to be a natural polymer material with good biocompatibility. Although barium sulfate has been widely used as a gastrointestinal contrast agent, its toxicity as a CS-Ba mixed material duct is unclear. Figure 5B shows that the cell survival rate of L929 cells treated with CS-Ba with different molecular weights was greater than 70%. Moreover, the cell survival rate of each group was significantly better than that of the positive control group (*P* < 0.05). Figure 5A shows that the number of cells increased rapidly over time, and the cells were in the full state. From the above results, it was proven that the CS-Ba ducts had no obvious cell cytotoxicity. Additionally, hemolysis is regarded as a primary and credible method of judging the blood compatibility of implants, and values up to 5% are permissible for biomaterials (Liu et al., 2017). If the material of a pancreatic duct stent cause hemolysis, since it is in direct contact with the blood around the anastomotic site, it will affect the blood supply of the anastomotic site tissue, thus affecting healing. In this study, the red blood cells incubated with different CS-Ba compound extracts showed no obvious hemolysis (a HR of almost 0), indicating that the CS-Ba compounds had good blood compatibility (Figure 5D).

To further explain the effects of the CS-Ba degradation products on the proliferation cycle of L929 cells, we quantitatively analyzed the cell proportions in each phase of proliferation by flow cytometry. From Figure 5C, we found that the number of cells in the G0/G1 phase was significantly lower in the three CS-Ba groups than in the control group, while the proportion of cells entering the S and G2/M phases in the three CS-Ba groups was higher, indicating that the CS-Ba extract contains ingredients that promote cell proliferation. Muzzarelli et al. (1988) believed that CS functions similarly to glycosaminoglycans because of its structural properties. As important glycoproteins in the extracellular matrix, glycosaminoglycans play an important role in cell proliferation, differentiation and morphogenesis. Thus, CS could promote the growth of fibroblasts in the anastomotic tissue and accelerate anastomotic healing after pancreatoenterostomy.

**FIGURE 6:**
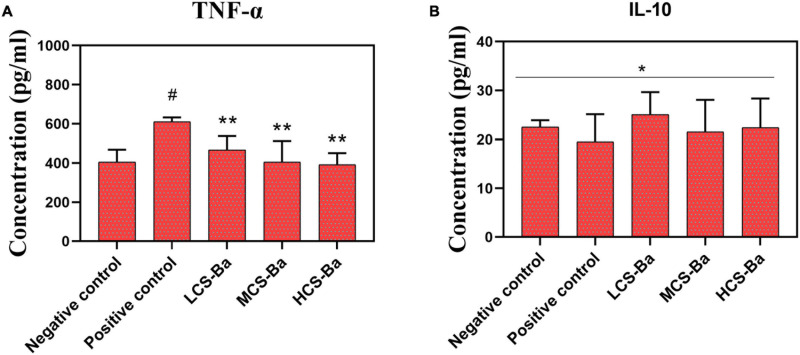
Different molecular weights of CS-Ba ducts induced RAW 264.7 macrophages to secrete **(A)** TNF-α and **(B)** IL-10 (* represents *P* > 0.05, ** represents *P* < 0.05 compared with the positive control; ^#^ represents *P* < 0.05 compared with the negative control).

Macrophages regulate tissue healing by secreting different inflammatory factors during the early and late stages of healing (Mirza et al., 2009; Rodero and Khosrotehrani, 2010; Koh and DiPietro, 2011; Mahdavian Delavary et al., 2011). In the early stage of healing, macrophages secrete proinflammatory factors (TNF-α, IL-1, and IL-6, etc.), proteases and reactive oxygen radicals to enhance the inflammatory response against pathogens at the damaged site. In the stage of tissue formation, macrophages secrete anti-inflammatory factors (IL-10, etc.) and phagocytic apoptotic cells to promote the regression of wound inflammation and initiate tissue repair. However, overexpression of inflammatory factors will disrupt the balance of the local immune environment and cause the overexpression of fibroblasts in the late stage of healing, leading to the occurrence of anastomotic stenosis. Therefore, the *in vitro* inflammatory factor induction experimental results were studied. As shown in Figure 6, we found that LCS-Ba, MCS-Ba and HCS-Ba did not induce an excessive inflammatory response in macrophages (*P* > 0.05).

**FIGURE 7:**
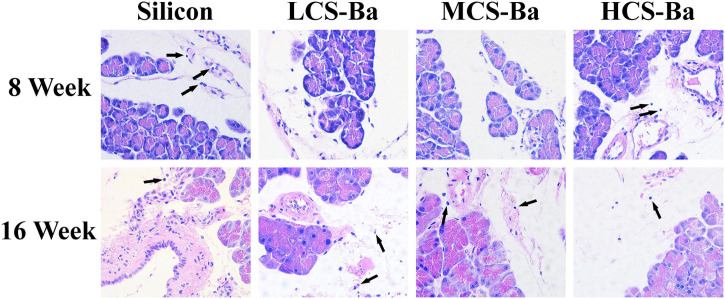
HE staining of the pancreas and surrounding tissues at the duct contact site at 8 and 16 weeks. There was no obvious abnormality in the pancreatic tissue, and few inflammatory cells were found (↑) (magnification 400×).

Rats have been widely used in animal studies of changes in acute pancreatitis (Gill et al., 1989). In animal experiments, CS-Ba ducts were fixed onto the surface of the pancreas in rats for evaluation so that they fully touched the pancreatic parenchyma. At the same time, we used the silicone tube for comparison, because it is an inert non-degradable material and is the most commonly used material for pancreatic duct stents. Serum amylase is the most sensitive hematological index in acute pancreatitis, and its changes mainly occur in the early stage. Therefore, rat serum amylase was tested in the first week after surgery. The results showed that except for a transient increase in amylase on the first day after the operation, the levels returned to near normal after 3 days (Supplementary Figure 1b). This result showed that the CS-Ba materials did not cause acute inflammation of the pancreas. Additionally, in the histological specimens at 8 and 16 weeks (Figure 7), pancreatic acinar cell vacuolation, fibrosis and granuloma were not found. However, a small amount of inflammatory cell infiltration was observed. This result does not reflect chronic toxicity of the material to the tissue. In addition, HE staining of the heart, liver, spleen, lung and kidney of the rats and blood biochemical indexes confirmed that CS-Ba did not have obvious toxicity to the important organs in the body after exposure for a long period of time (Supplementary Figures 1c–f, 2).

### Mechanical Properties of CS-Ba Ducts and Their Matching With Soft Tissue

**FIGURE 8:**
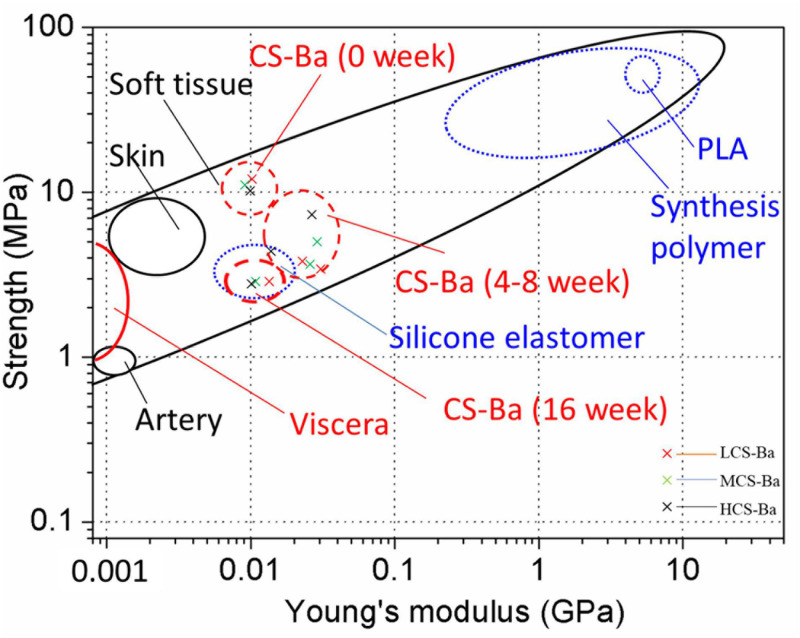
Strength and Young’s modulus of CS-Ba ducts during degradation in SPJ solution compared with viscera and other biopolymers.

Mechanical properties are key factors that affect tissue regeneration in addition to biocompatibility. Matching of the Young’s modulus of the implant material with that of the tissue can reduce complications such as tissue damage, inflammation, and necrosis and contribute to cell adhesion and tissue regeneration (Lee et al., 2019). Figure 8 shows the comparison between the mechanical properties of CS-Ba in the degradation process with other polymers and soft tissues (Ashby, 2011; Jang et al., 2018; Liravi and Toyserkani, 2018). The elastic modulus and strength of soft tissues are generally low, as the Young’s modulus of human internal organs is lower than 0.001 GPa. Currently, a silicone rubber stent is commonly used for pancreaticojejunostomy, and it has a Young’s modulus of 0.006–0.02 GPa. Compared with other degradable polymers, such as PLA (Young’s modulus of 2–4 GPa and strength of 40–70 MPa), the Young’s modulus of silicone rubber is more suitable for the repair of soft tissues such as the pancreas, but its inability to degrade limits its further applications. In this study, the Young’s modulus of CS-Ba increased in the early stage and then decreased but was still 0.01 GPa after 16 weeks, which is close to the modulus of the human viscera. During degradation, the strength continued to decline; however, it could be maintained at 3–4 MPa after 16 weeks, which represents a good supportive effect compared with internal organs. It could prevent the anastomotic site from collapsing and causing stenosis. In this study, the original Young’s modulus of three molecular weights of CS-Ba ducts was approximately 8–10 MPa, which is slightly lower than the mechanical properties of the pure CS previously studied (Zhao et al., 2020). This is because the blended system of the polymer and heavy metal salt cannot guarantee fusion of the two phases and is prone to incompatibility or two-phase separation, which will become a source of fracture and affect the mechanical properties of the final material (Rawls et al., 1990). At the 16th week of degradation, the tensile strengths and Young’s modulus of the CS-Ba ducts were basically the same as those of silicone elastomers, which indicated that after 16 weeks of degradation of CS-Ba ducts, their mechanical properties maintained sufficient support for the drainage of pancreatic fluid, and no corresponding collapse occurred.

**FIGURE 9:**
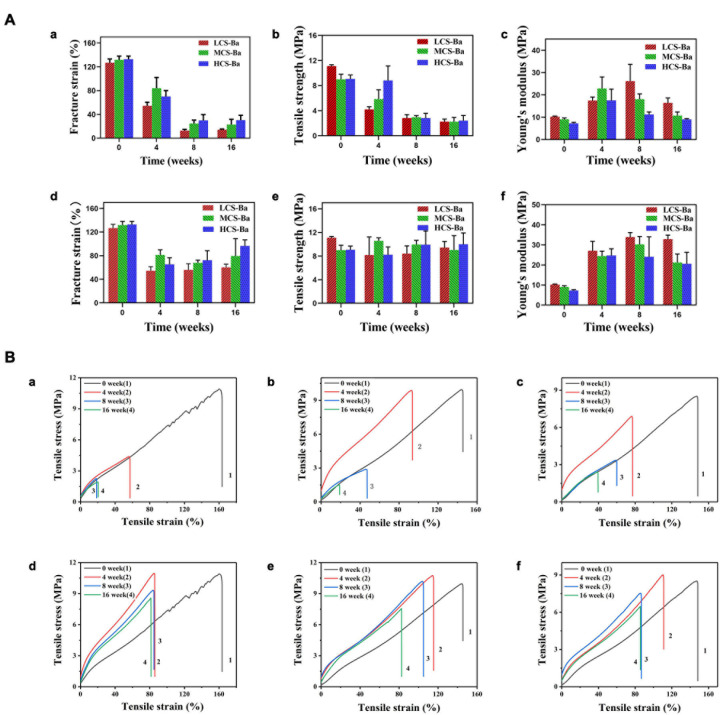
Tensile properties of CS-Ba rods manufactured with different molecular weights. **(A)** Fracture strain, tensile strength, and Young’s modulus. [(a–c) In SPJ solution with pancreatin. (d–f) In SPJ solution without pancreatin]. **(B)** Tensile stress and strain curves. [(a, d) LCS-Ba rods. (b, e) MCS-Ba rods. (c, f) HCS-Ba rods.].

An interesting phenomenon appeared in this study. As shown in Figure 9A, in SPJ solution, the breaking strain and tensile strength of CS-Ba with different molecular weights decreased with increasing degradation time, and the lower the molecular weight was, the faster was the rate of decline (*P* < 0.05). In the SPJ solution without pancreatin, the fracture strain decreased in the early stage but did not decrease further after 4 weeks. There were no obvious changes in the tensile strength, indicating that the degradation of the material was not obvious in the alkaline solution. Interestingly, the Young’s modulus of the materials increased in the early stage but decreased in the late stage in the SPJ group containing pancreatin for the different molecular weights of CS-Ba samples. In the SPJ solution without pancreatin, the modulus remained unchanged after the initial increase and did not continue to decrease. This change rule can be clearly seen in the stress-strain curve (Figure 9B). This result was similar to the change trend of the water absorption rate of the material (Supplementary Figure 3). In the early stage, the water absorption rate decreased, and the material became hard. As degradation progressed, the water absorption rate slowly increased. This phenomenon also appeared during the degradation process of electrodeposited CS catheters in Nawrotek et al.’s (2020) study. This may be due to the deprotonation of the CS amine groups in the early stage under alkaline conditions, the reduction in ion repulsion, and the strengthening of molecular connections through hydrogen bonds and hydrophobic interactions, making the texture of the material harder, increasing the Young’s modulus, and decreasing the water absorption rate. In the solution with pancreatin, as the degradation progressed, cracks or holes appeared on the surface of the material, allowing water to enter the inside of the material, causing the water absorption rate to increase and the modulus to decrease. In the alkaline solution without pancreatin, the tensile strength of CS-Ba did not change significantly, while the Young’s modulus increased and did not decrease in the later stage. The results confirmed that pancreatin can accelerate the degradation of CS-Ba.

### Cs-Ba Ducts With Good Radiopaque Properties

**FIGURE 10:**
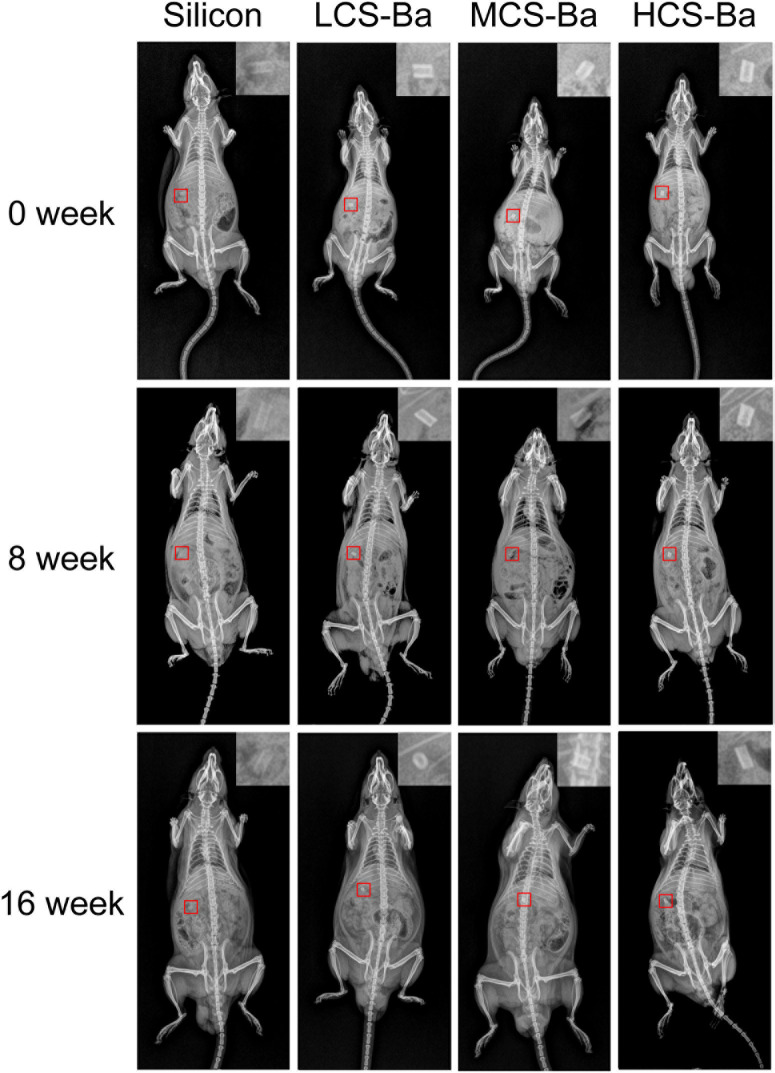
Radiopaque performance of CS-Ba ducts with different molecular weights during the degradation process *in vivo.*

In most polymer materials, the macromolecular chain only contains elements with a low electron density and low specific gravity such as C, H, O, and N. There are no elements with a high electron cloud density (such as halogens or metal elements), so the materials cannot be radiographically tested. To overcome this inherent defect, traditional polymer molecules must be endowed with radiation-impermeable properties to meet medical needs. In this study, degradable radiopaque CS-Ba ducts were prepared so that the ducts could be detected by X-ray after implantation. Nordback et al. (2012) verified that PLA stents mixed with 23% barium sulfate have good radio-opacity effects. From Figure 10, X-ray observations indicated that the imaging effects of the ducts containing 23% barium sulfate were significantly better than those of the silicone rubber tube. With increasing implantation time, the duct radiopaque status was slightly decreased compared with the initial status, and the peripheral duct edge in the LCS-Ba group became blunt, but the outline was still not deformed, indicating that the CS-Ba ducts can maintain a certain radial support force after 16 weeks. In addition, the imaging performance was good, which is very beneficial for future observations and follow-up after the material is implanted in the human body.

As mentioned above, this study investigated the process, microstructure and performance of CS-Ba ducts for pancreaticojejunostomy tissue repair. The microstructure of the CS-Ba ducts was analyzed by SEM, FTIR, and XRD. The tensile strength, Young’s modulus and fracture strain of CS-Ba ducts were measured to evaluate the degree of mechanical matching to pancreatic tissue. The degradation performance and biocompatibility of CS-Ba ducts of different molecular weights in SPJ *in vitro* and *in vivo* were also studied. The radioopacity of the CS-Ba ducts in the body was evaluated. There are still some shortcomings in this study. The best degradation time of the pancreatic duct stent is approximately 3–6 months, but the degradation rate of 50,000 g/mol molecular weight chitosan in this study was still unable to meet its needs. Therefore, in future work, we will try to further reduce its molecular weight to regulate the degradation time of the stent and use large animals, such as pigs, for further verification.

## Conclusion

This study verified the advantages of extrusion-based 3D printed CS-Ba ducts as pancreatic duct stents. The following conclusions can be drawn:

1. CS-Ba ducts prepared by this method have the following advantages: compact and ordered structure, good molding effects, and a simple preparation method. The length and diameter of the CS-Ba ducts can be adjusted to customize personalized pancreatic duct stents for the patient.
2. CS-Ba ducts possess mechanical properties that match those of soft tissues, and they can meet the degradation time required for the healing of the pancreaticojejunostomy by controlling the molecular weight of CS. In an *in vitro* study, it was shown to have the potential to promote the growth of fibroblasts, which could accelerate anastomotic healing after pancreatoenterostomy.
3. The CS-Ba ducts had a good radiopaque performance and could be detected by X-ray after surgery. In conclusion, extrusion-based 3D-printed CS-Ba ducts can feasibly replace silicone rubber tubes as pancreatic duct stents and have considerable application potential in the future.

## Data Availability Statement

The raw data supporting the conclusions of this article will be made available by the authors, without undue reservation.

## Ethics Statement

The animal study was reviewed and approved by the Experimental Animal Ethical Committee of Fujian Medical University.

## Author Contributions

MP: conceptualization, investigation, methodology, formal analysis, software, writing – original draft, and writing – review and editing. CZ: data curation, validation, methodology, and formal analysis. ZX and YY: investigation. TT: project administration. JL: funding acquisition and conceptualization. HH: funding acquisition, supervision, and writing – review and editing. All authors contributed to the article and approved the submitted version.

## Conflict of Interest

The authors declare that the research was conducted in the absence of any commercial or financial relationships that could be construed as a potential conflict of interest.
