# Supplementary material for: Radiopaque Chitosan Ducts Fabricated by Extrusion-Based 3D Printing to Promote Healing After Pancreaticoenterostomy
Source: Front Bioeng Biotechnol. 2021 Jun 4;9:686207. doi: 10.3389/fbioe.2021.686207 (PMC8212045; doi:10.3389/fbioe.2021.686207)
Supplement: Supplementary file 1 [file Data_Sheet_1.docx]

Supplementary Material

# Supplement- Figures

# S-1 The general health status of the animals after surgery

# Within 7 days after duct implantation, the rats ate normally, wound healing was good, and no fatal accidents occurred, and significant weight was gained within 16 weeks after the operation (S-Fig. 1a). On days 1, 3, and 7 after the materials were implanted into the rats, we measured the values of rat serum amylase to observe whether the materials could cause acute injury to the pancreas. As shown in S-Fig. 1b, preoperative serum amylase was used as the normal reference value, and it was found that the levels of serum amylase in the CS-Ba and control groups increased on the first day after surgery and then began to decline, dropping to near normal on the third day. The initial increase was considered to be transient due to intraoperative injury to pancreatic tissue.

#
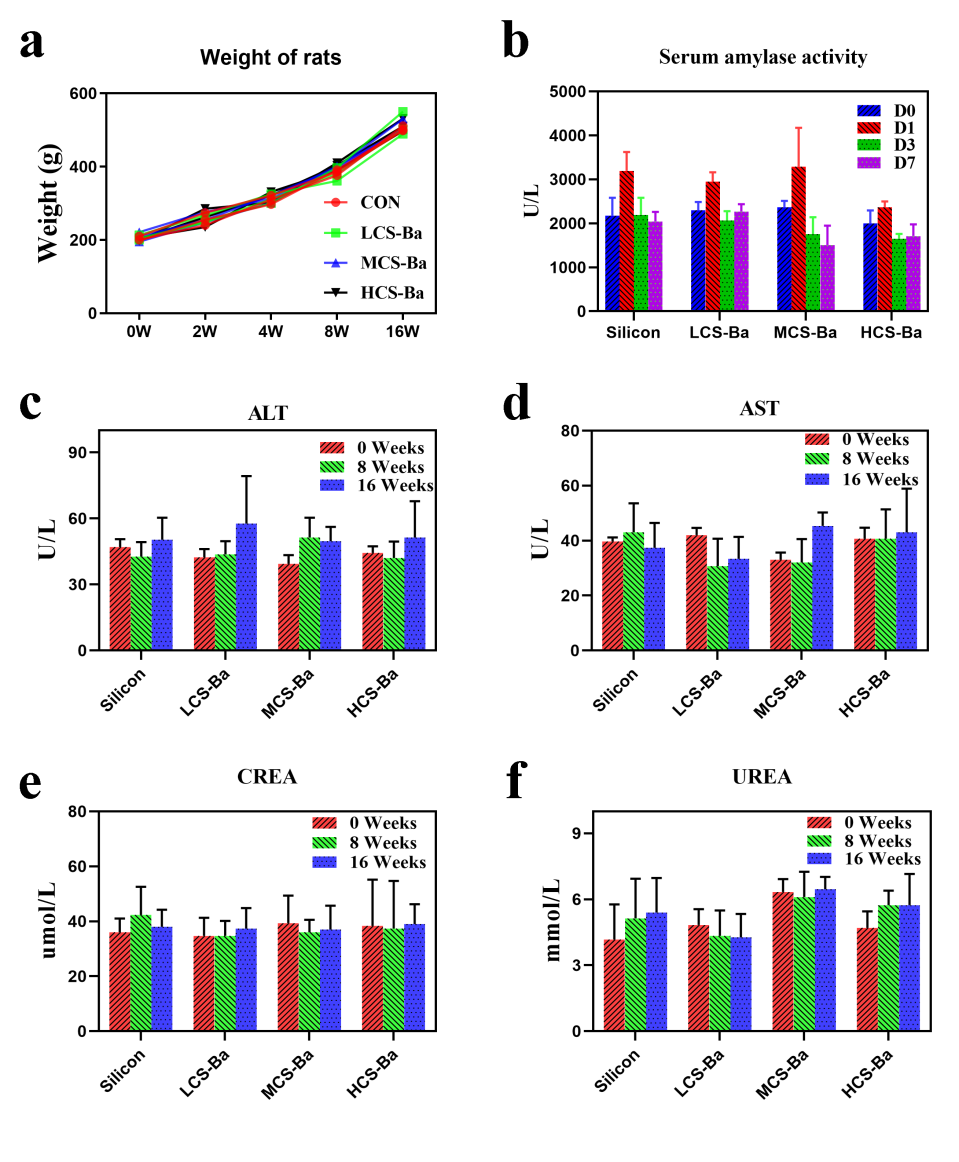


# S-Fig. 1 Health status and blood biochemical test of rats after surgery. (a) Rat weight changes after surgery. (b) Changes in serum amylase in rats before surgery and 1, 3, and 7 days after the operation. (c-f) Postoperative liver and kidney function in rats (including alanine transaminase, aspartate aminotransferase, creatinine, and [urea](javascript:;) [nitrogen](javascript:;)).

# S-2 [Histocompatibility](javascript:;)

# We evaluated whether the CS-Ba duct degradation products were toxic to various rat organs after being absorbed and metabolized in the body during long-term implantation. As shown by the HE staining in S-Fig. 2, there was no obvious pathological tissue damage or abnormality in the various organs (heart, liver, spleen, lung, and kidney) of SD rats in the different [molecular](javascript:;) [weight](javascript:;)s of CS-Ba implantation groups. In addition, as shown in S-Fig. 1c-f, various biochemical indicators, such as alanine transaminase, aspartate aminotransferase, creatinine, and [urea](javascript:;) [nitrogen](javascript:;), were within the normal ranges. These results indicated that the degradation products of CS-Ba did not cause damage to the important organs of the rats.

#
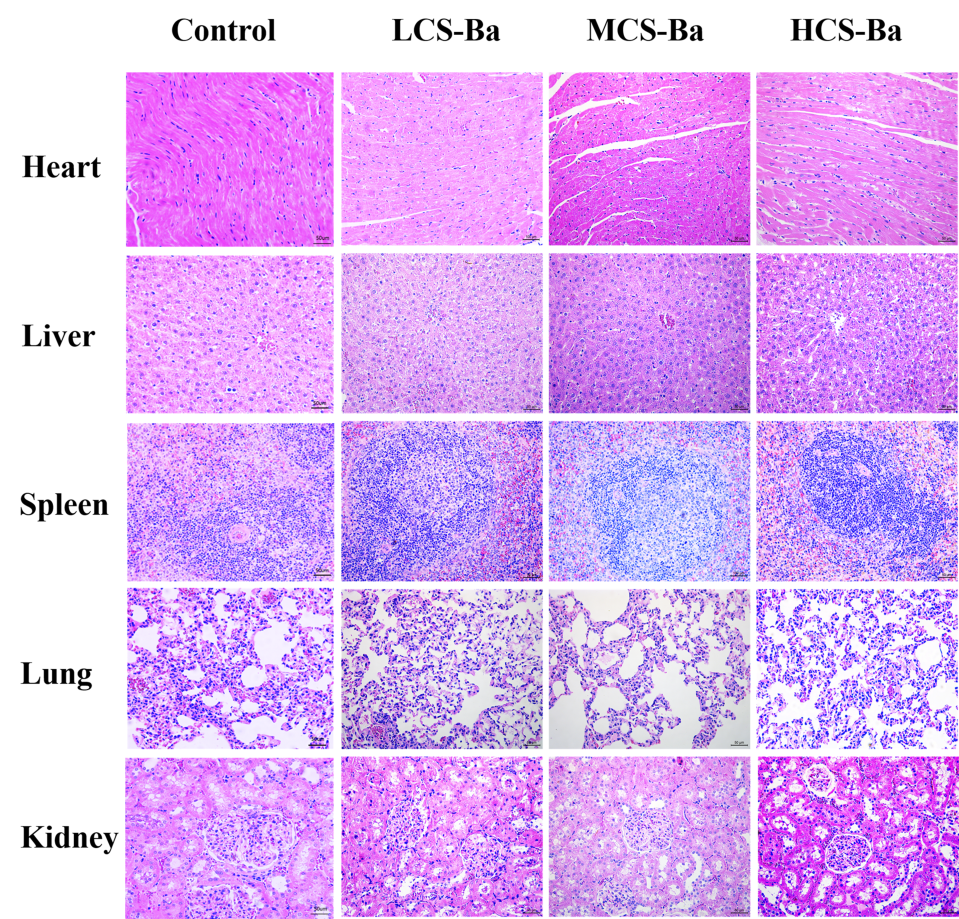


# S-Fig. 2 Pathological changes in the main organs were evaluated by HE staining in the 16th week. As shown in the figure, the ducts in each group did not cause obvious pathological changes (magnification 200×).

# S-3 Water absorption

# As shown in S-Fig. 3, in the SPJ solution containing pancreatin, the water absorption rate of each group decreased significantly during the early stage (T≤4 weeks) and began to rise after 4 weeks. By the 16th week, the water absorption rates of LCS-Ba, MCS-Ba and HCS-Ba were 50.61, 52.57 and 53.65%, respectively, showing no significant difference (P > 0.05). However, in the SPJ solution without pancreatin, the water absorption rate of each group progressively decreased. At the 16th week, the water absorption rates of LCS-Ba, MCS-Ba, and HCS-Ba were 37.50, 36.51, and 40.05%, respectively, and were not significantly different (P > 0.05). However, at 8 and 16 weeks, the water absorption rates of the stents in the SPJ solution containing pancreatin were significantly higher than those in the SPJ solution without pancreatin (P < 0.05).

#
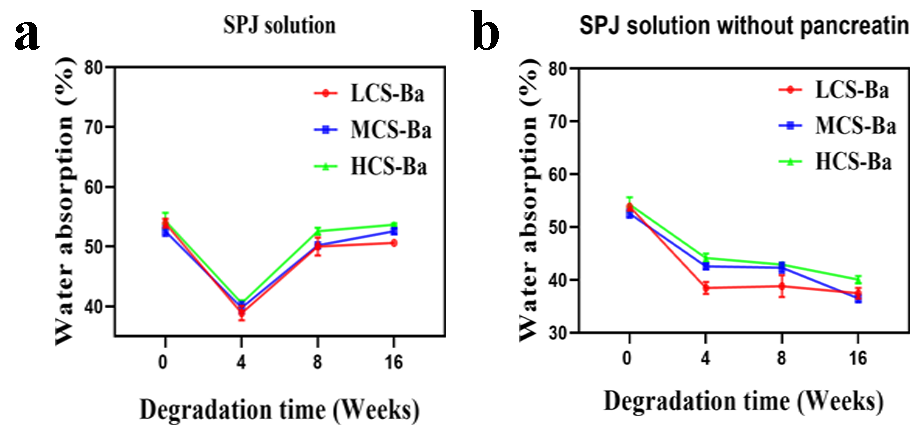


# S-Fig. 3 Water absorption trend of the CS-Ba ducts in SPJ solution with pancreatin (a) and without pancreatin (b).
